# Preparing for the Implementation of Long-Acting Injectable Cabotegravir for HIV Pre-Exposure Prophylaxis Within the Brazilian Public Health System (ImPrEP CAB Brasil): Qualitative Study

**DOI:** 10.2196/60961

**Published:** 2024-10-24

**Authors:** M Cristina Pimenta, Thiago Silva Torres, Brenda Hoagland, Mirian Cohen, Claudio Gruber Mann, Cristina M Jalil, Eduardo Carvalheira, Lucilene Freitas, Nilo Fernandes, Debora Castanheira, Marcos Benedetti, Julio Moreira, Keila Simpson, Roberta Trefiglio, Gabrielle O’Malley, Valdilea G Veloso, Beatriz Grinsztejn

**Affiliations:** 1 Instituto Nacional de Infectologia Evandro Chagas, Fundação Oswaldo Cruz Rio de Janeiro Brazil; 2 Grupo Arco-Íris de Cidadania LGBT Rio de Janeiro Brazil; 3 Associação Nacional de Travestis e Transexuais Salvador Brazil; 4 Department of Global Health, Schools of Medicine and Public Health, University of Washington Seattle, WA United States

**Keywords:** pre-exposure prophylaxis, PrEP, implementation, public health system, cabotegravir, HIV prevention, Latin America, long-acting PrEP

## Abstract

**Background:**

Although long-acting, injectable cabotegravir (CAB-LA) pre-exposure prophylaxis (PrEP) has proven efficacious for HIV prevention in clinical trials, research is needed to guide effective implementation in real-world settings. Formative work with community members and health care providers (HCPs) is important to provide insight into the needs and contexts of specific populations and reveal potential barriers and facilitators for implementation projects.

**Objective:**

We aimed to describe the results from formative work to develop an implementation package for CAB-LA PrEP within the ImPrEP CAB Brasil study.

**Methods:**

ImPrEP CAB Brasil is an implementation study of same-day delivery of CAB-LA PrEP for young sexual and gender minority (SGM) groups (aged 18-30 years) in 6 existing oral PrEP public health clinics. We conducted formative research to prepare for the implementation of ImPrEP CAB Brasil through community mobilization, process mapping with HCPs with experience in CAB-LA, and focus group discussions (FGDs) with young SGM groups (n=92) and HCPs (n=20) to identify initial perceptions of facilitators and barriers for CAB-LA PrEP implementation, refine the mobile health (mHealth) educational tool, and evaluate the acceptability of using a text message appointment reminder intervention through WhatsApp. FGDs were recorded, transcribed, systematically coded, and analyzed with thematic categorization by trained researchers using a qualitative data analysis program ATLAS.ti (version 7).

**Results:**

A community mobilization team comprising 34 SGM community leaders collaborated in creating a prototype for an mHealth educational tool and contributed to the planning of peer education activities. We created 3 process maps for each site to describe the initial visit, follow-up visits, and laboratory flow. The main challenge identified for same-day CAB-LA PrEP delivery was the extended duration of clinic visits due to the numerous laboratory tests and HIV counseling steps required. Proposed solutions included having point-of-care HIV rapid tests instead of laboratory tests and additional counseling staff. Barriers for CAB-LA PrEP implementation identified through FGDs were the training of HCPs, support for adherence to injection appointments, and stigma or discrimination against SGM groups and persons using PrEP. The mHealth educational tool and WhatsApp reminders were highly acceptable by SGM groups and HCPs, indicating their potential to support PrEP choice and adherence. Content analysis on the cultural appropriateness of the language and overall clarity of the material contributed to the refinement of the mHealth tool.

**Conclusions:**

Structured formative work with SGM persons and HCPs generated important refinements to context-specific materials and plans to launch ImPrEP CAB Brasil in public health clinics. Ongoing implementation monitoring will use the process maps to identify additional barriers and potential solutions to same-day delivery of CAB-LA PrEP. Summative evaluations are needed to measure the effectiveness of the mHealth educational tool to support PrEP choice and the use of WhatsApp appointment reminders.

## Introduction

### Background

Sexual and gender minority (SGM) groups are disproportionally affected by the HIV epidemic in Brazil. While HIV prevalence in the overall Brazilian population was estimated at approximately 0.4% [1], the prevalence was estimated at 23% among gay, bisexual, and other men who have sex with men [2] and at 30% among transgender women [3,4]. SGM participants aged 15-30 years are particularly susceptible to HIV infection [5]. A recent study estimated annualized HIV incidence in Brazil among SGM groups not using pre-exposure prophylaxis (PrEP) at 2.62% (95% CI 2.14%-3.10%), with estimates of 3.48% (95% CI 2.51%-4.45%) and 3.65% (95% CI 2.34%-4.18%) among SGM participants aged 18 to 24 and 25 to 30 years, respectively [6].

Oral PrEP has been effective in reducing population-level HIV incidence in multiple settings [7-11]. It has been available through the national public health system (Sistema Único de Saúde [SUS]) in Brazil since 2017 with no direct cost to users [12]. As of August 31, 2024, there were 1051 health facilities providing oral PrEP to 99,983 persons [13]. However, among those who initiated PrEP in the Brazilian SUS, 32% discontinued in the past 12 months (no refill of PrEP pills); discontinuation rates were higher among those aged 15 to 17 years (60%) and 18 to 24 years (41%) [13]. In the ImPrEP study, the largest oral PrEP implementation study conducted in Latin America, young SGM groups from Brazil, Mexico, and Peru had higher HIV incidence and lower oral PrEP adherence compared to their older counterparts [14,15]. Long-acting PrEP agents, which do not require daily or planned oral dosing, may mitigate the risk of HIV acquisition among young SGM persons.

Long-acting, injectable cabotegravir (CAB-LA) has proven efficacious for HIV prevention in phase 3 clinical trials (HIV Prevention Trials Network [HPTN] 083 and HPTN 084) [16,17]. Data from these studies led to the recommendation of CAB-LA for HIV prevention by the World Health Organization in July 2022 [18]. In Brazil, CAB-LA for HIV prevention has regulatory approval, but this strategy is not yet incorporated in the Brazilian PrEP Guidelines and is not available through the public health system. Strong formative research with community members and health care providers (HCPs) will increase the likelihood that the delivery of CAB-LA PrEP will be effective in routine health service settings [19,20].

### Objectives

The ImPrEP CAB Brasil implementation study aims to generate evidence to inform national policies and program implementers about how to provide injectable CAB-LA PrEP for young SGM participants aged 18 to 30 years within the Brazilian SUS. The main implementation objectives are to describe facilitators and barriers to integrating CAB-LA into oral PrEP services, evaluate whether a mobile health (mHealth) educational tool optimizes PrEP decision-making, and determine whether reminders via WhatsApp improve adherence to CAB-LA clinical appointments. This paper describes our formative qualitative work to develop an initial implementation package for CAB-LA PrEP within the ImPrEP CAB Brasil study.

## Methods

### ImPrEP CAB Brasil Study

ImPrEP CAB Brasil is a type 2 hybrid effectiveness-implementation open-label cohort study with a convergent mixed methods approach (quantitative and qualitative) [21]. The study participants from the implementation study will include young PrEP-naive SGM adults aged 18 to 30 years (gay, bisexual, and other cisgender man who have sex with men [cisgender MSM]; transgender persons; nonbinary persons; and other gender fluid identities) in 6 Brazilian cities: Rio de Janeiro, São Paulo, Manaus, Salvador, Florianópolis, and Campinas. Details of the study protocol are described elsewhere [22]. In brief, individuals seeking PrEP will be exposed to education and counseling about HIV prevention, including oral and injectable PrEP (standard of care), or to an mHealth education and decision support intervention in addition to standard of care (in a 1:1 allocation ratio). Then, participants who choose injectable PrEP (N=1200) will be enrolled and receive the CAB-LA injection on the same day and will be followed up for 52 weeks; half of the participants will be randomized to receive a digital appointment reminder via WhatsApp.

### Ethical Considerations

This study was reviewed and approved by the Evandro Chagas National Institute of Infectious Diseases–Fiocruz institutional review board (CAAE 59166522.7.1001.5262), World Health Organization Research Ethics Review Committee, and local institutional review boards at each site. All participants provided written informed consent (no illiterate participants were involved in this study). Data were de-identified before analysis and participants received transportation reimbursement. All methods were carried out in accordance with relevant guidelines and regulations. This study was registered at ClinicalTrials.gov (NCT05515770) on August 29, 2022.

### Procedures of Formative Work

#### Community Mobilization

Community mobilization has been a pivotal element in the success of oral PrEP implementation and demand creation across a plurality of SGM groups [23]. Here, we briefly describe the community mobilization efforts in preparation for the ImPrEP CAB Brasil study. We invited SGM community leaders from each study site and lesbian, gay, bisexual, transgender, queer, intersex, asexual, agender, pansexual and nonbinary persons (LGBTQIAPN+) from nongovernmental organizations (NGOs) to (1) participate with researchers and HCPs in the ImPrEP CAB Brasil project launch event, (2) provide input for the development of the prototype video-based mHealth educational tool, (3) assist in developing community mobilization strategies for the ImPrEP CAB Brasil study, and (4) establish a community advisory board for the ImPrEP CAB Brasil study to serve as community partners and advocates for PrEP accessibility and to support the interpretation of interim findings for the wider community.

#### Process Mapping

Process maps are a diagrammatic representation of organizational processes and are widely used in health care quality management and improvement. However, the literature is scarce in describing their use to incorporate clinical innovations into routine clinical practice. Using participatory design methods, we developed process maps with each participating site using a 4-step approach (preparation, mapping or analyzing, customization or cocreation, and validation) to identify and mitigate possible service delivery bottlenecks. We conducted individual interviews with HCPs at each study site to create initial process maps. We then held web meetings with additional HCPs and peer educators from each site who were encouraged to provide honest and constructive opinions about the proposed maps and possible barriers and risks to planned models of service delivery. Finally, we asked the same individuals to validate revised process maps and to ensure there were no missing steps. We used Bizagi Modeler Software (version 4.0) to create the process maps.

#### Focus Group Discussions

We conducted focus group discussions (FGDs) with SGM groups and HCPs with three main objectives: (1) to understand initial perceptions of facilitators and barriers about the proposed implementation of same-day CAB-LA PrEP, (2) to seek feedback for the refinement of the prototype video-based mHealth educational tool, and (3) to assess acceptability of text messages through WhatsApp (the most common messaging app used in Brazil) for appointment reminders. SGM groups were recruited at each study site and community organizations through informal invitations. FGDs with HCPs were conducted at 2 sites (Rio de Janeiro and São Paulo) that had prior experience in a CAB-LA clinical trial (HPTN 083). SGM participants were invited by peer educators from the oral PrEP services. The eligibility criteria included SGM participants aged 18 to 30 years who self-identified as cisgender MSM, transgender, or nonbinary person and HCPs aged ≥18 years who provided care to persons receiving CAB-LA during the HPTN 083 study. Exclusion criteria included persons unwilling and unable to provide written informed consent. Eligible participants signed informed consent forms before FGD initiation, with no refusal to participate during or after the consent process.

We developed 2 FGD guides for this study: one for HCPs and one for SGM persons. We used these guides to generate discussion about the mHealth tool and WhatsApp messages and to elicit general perceptions of what would be needed to integrate injectable PrEP delivery to existing oral PrEP services. To refine the mHealth tool, participants were presented with the mHealth prototype which was comprised of 5 short videos (2-3 minutes) with characters representing diverse genders and racial groups, designed to engagingly convey information on oral and injectable PrEP and HIV combination prevention, followed by discussion about the comprehension and acceptability of the mHealth prototype. Proposed WhatsApp messages were also presented to participants, followed by discussion about the acceptability of WhatsApp text messages as reminders for injection appointments and on the content of specific messages. The discussion guide for HCPs also elicited perceptions of major challenges related to PrEP implementation based on their experience with PrEP services.

Data collection was conducted through FGDs in which interaction is an integral part of the method. In the process, group meetings allow participants to explore their perceptions and points of view based on reflections about presented themes. The responsibilities of the FGD team were well defined, and interviewers had no relationship with FGD participants. The HCPs were interviewed by researchers with no past involvement in the HPTN 083 clinical trial. All FGDs were conducted in Portuguese, recorded, and transcribed.

The FGD data were organized based on the preestablished concepts of the FGD guides for each population group. Consistency and precision were ensured under the coordination of a team of 6 experienced researchers trained in qualitative methodologies. Data organization included the triangulation of the audios, the transcribed material, and the observational reports of all FGDs. Transcribed texts were reviewed and validated by FGD researchers before categorization, coding, and analysis. Code labeling and units of analysis were identified by iteratively examining the texts and isolating self-contained segments that capture a singular idea, insight, or piece of information.

We used content analysis with thematic categorization and systematic coding according to Bardin [24]. The primary aim of the content analysis is to deliver a comprehensive and nuanced understanding of the data gathered from the FGDs, aligning with the study's objectives. Therefore, qualitative insights are prioritized in this approach. The researcher team deductively established a set of 6 main categorical codes (challenges, facilitators, language, format, content, and acceptability) and 3 axial codes (implementation of CAB-LA, evaluation of an mHealth tool, and WhatsApp messages) based on review of the FGD guide. Additional inductive codes were developed through multiple reviews of the transcripts. Researchers reconciled presented differences in interpretation during the synthesis and analysis of the texts. Codes and transcripts were migrated to ATLAS.ti Scientific Software Development GmbH (version 7.5.18) in Portuguese to aid data management, pattern identification, relationships, and themes through simultaneous collaborative work. This iterative process ensured a comprehensive understanding of the data, allowing for interpretation that accurately reflects the participants’ perspectives. By engaging in thorough discussions and deliberations, the research team was able to acquire richer insights and enhance the reliability and validity of the findings. The final codebook was consolidated and used to code all transcripts. Coded text was analyzed by population group (SGM groups and HCPs) and organized into themes [25-27].

## Results

### Community Mobilization

During the project launch event on October 4 to 5, 2022, community mobilization and involvement was initiated. The study team presented the ImPrEP CAB Brasil protocol to 34 SGM community leaders allowing them to forefront their needs and concerns. The study team supported the SGM community leaders with study information needed to engage important stakeholders, including information they could use in a broader community mobilization effort to ensure a more inclusive and diverse composition of SGM groups for the study. These leaders also collaborated in developing the prototype for the study’s mHealth educational tool, participated in study protocol training sessions, and contributed to the planning of peer education activities.

Community mobilization activities also included discussions of technical and operational study content focusing on the following points: (1) peer educators’ intervention approaches; (2) ideas for project-related graphic materials and visual identity; and (3) pertinent information to be disseminated, such as characteristics of each PrEP modality, side effects, and frequency of use. As a result of these discussions, we included 12 SGM participants (2 at each site) as peer navigators and educators at the study clinics. The peer navigators will promote a welcoming and safe environment, build trust between researchers and participants, raise awareness of HCPs to address language barriers, leverage community resources for specific social needs of participants, and promote their linkage and retention to PrEP.

The study community advisory board was established, composed of 2 members from each participating city (n=12). The community advisory board is coordinated by 1 transgender woman and 1 gay man who are leaders of an LGBTQIAPN+ NGO and national key stakeholders.

### Process Mapping

From July 27 to November 30, 2022, 3 process maps for each site were created to describe the initial visit, follow-up visits, and laboratory flow (18 maps total) for the implementation of injectable PrEP services using a model where the first injection is given the same day as the HIV and laboratory screening. Multimedia Appendix 1 shows the part of the process map of the initial PrEP visit at the Instituto Nacional de Infectologia Evandro Chagas–Fiocruz site.

The main anticipated barrier and risk identified during process mapping was the extended duration of visits given that a high number of laboratory and HIV counseling steps are necessary for same-day PrEP delivery. Proposed solutions included point-of-care HIV rapid tests instead of laboratory tests and training HCPs on their proper use. Sites also reinforced the need to increase the number of professionals to perform counseling. To better serve the study population, 2 sites identified the need to expand clinic hours during the implementation study for those who cannot seek services during usual clinic hours. All sites validated the revised process maps and used them to organize a checklist to facilitate study preparedness.

### Focus Group Discussions

A total of 14 FGDs were conducted between April 11 and June 14, 2023. Each FGD lasted between 50 and 60 minutes. A total of 12 FGDs were conducted with young SGM aged 18 to 30 years, with 2 FGDs at each of the 6 study sites. Each site had 1 FGD with cisgender men and 1 FGD with transgender persons. Nonbinary persons were free to choose to participate in the group in which they felt most comfortable. Three nonbinary persons preferred to join the cisgender men group and 3 joined the transgender persons group in different sites. One FGD with HCPs (nurses, psychologists, pharmacists, and medical physicians) was conducted at the 2 study sites that had participated in the HPTN 083 clinical trial. In total, 20 HCPs and 92 SGM persons participated in the FGDs (Table 1). There were no refusals to participate during or after the consent process. The results by population group are discussed in subsequent sections.

**Table 1:** Characteristics of participants included in the qualitative assessment.

Characteristics			Total (N=112), n (%)		SGM^a^ persons (n=92), n (%)		HCPs^b^ (n=20), n (%)
**City**							
	Campinas	13 (11.8)		13 (14.1)		0 (0)	
	Florianópolis	13 (11.8)		13 (14.1)		0 (0)	
	Manaus	13 (11.8)		13 (14.1)		0 (0)	
	Rio de Janeiro	29 (26.5)		20 (21.7)		9 (45.5)	
	Salvador	19 (17.3)		19 (20.6)		0 (0)	
	São Paulo	25 (22.7)		14 (15.2)		11 (54.5)	
**Gender**							
	Cisgender men	41 (36.6)		39 (42.4)		2 (10)	
	Cisgender women	16 (14.3)		0 (0)		16 (80)	
	Nonbinary	6 (5.4)		6 (6.5)		0 (0)	
	Transgender men	3 (2.7)		3 (3.3)		0 (0)	
	Transgender women	26 (23.1)		25 (27.2)		1 (5)	
	*Travesti* ^c^	20 (17.9)		19 (20.6)		1 (5)	
**Race**							
	Asian	2 (1.8)		1 (1.1)		1 (5)	
	Black	33 (0.3)		31 (33.7)		2 (10)	
	Indigenous	5 (4.5)		5 (5.4)		0 (0)	
	*Pardo* ^d^	31 (28.2)		25 (27.2)		6 (30)	
	White	41 (37.3)		30 (32.6)		11 (55)	
**Education**							
	Primary incomplete	5 (4.5)		5 (5.4)		0 (0)	
	Primary complete	1 (0.9)		1 (1.1)		0 (0)	
	Secondary incomplete	6 (5.5)		6 (6.5)		0 (0)	
	Secondary complete	32 (29.1)		31 (33.8)		1 (5)	
	Tertiary incomplete	29 (26.5)		27 (29.3)		2 (10)	
	Tertiary complete	39 (35.5)		22 (23.9)		17 (85)	

^a^SGM: sexual and gender minority.

^b^HCP: health care provider.

^c^In Latin America, *travesti* is a term for people who were assigned male at birth but identify as feminine.

^d^The term *pardo* is used in the Brazilian census to indicate multiracial individuals.

### Health Care Professionals

#### Perceptions About Implementation of Injectable PrEP

HCPs participating in the FGDs emphasized the importance of engaging a multidisciplinary staff team for successful integration of CAB-LA PrEP into existing public health oral PrEP services (PrEP-SUS), including physicians, nurses, staff for screening and monitoring of persons using injectable PrEP, psychologists for counseling, and administrators. Clinical assessment was considered essential before initiation of injectable PrEP due to the specific requirements of the medication, such as formulation, long-acting effect, and intramuscular administration. The presence of psychologists and peer educators was considered particularly important to guarantee emotional support and a hospitable and embracing environment as some individuals may experience discomfort and pain during the injection. Furthermore, recognizing that young SGM persons often encounter heightened challenges in HIV and sexually transmitted infection (STI) prevention, participants emphasized the importance of the study team to be prepared to address both the clinical and psychosocial aspects of their needs effectively. They highlighted the necessity for a person-centered approach that not only focuses on the medical interventions but also provides emotional support for risk assessment when choosing between oral or injectable PrEP and resources to navigate the unique obstacles faced by this population. This comprehensive strategy is seen as essential for fostering a supportive environment and promoting well-being among young SGM individuals:

The population we want to access today, both transgender persons and cis MSM aged 18 to 30 years, sex workers, have very important psychosocial needs. Most of the people currently coming in for oral PrEP are young. During HIV diagnoses they always bring a very high emotional load. So, you can’t think about not having a supportive approach with a social worker, a psychologist or counselor to facilitate linkage and retention [to PrEP service]. If you focus only on the PrEP medication, you will soon lose that patient or study volunteer. At four, six months, you won’t access them anymore.

Cisgender woman, aged 45 to 50 years

The retention of younger SGM participants in PrEP services was anticipated as a major challenge, particularly in health facilities in more underserved contexts. Thus, it was recommended that study clinics plan for situations in which people do not return for injection appointments within the appropriate window. The importance of improving standard follow-up and support services and developing measures to encourage adherence was emphasized. Other possible barriers were related to a lack of adequate supplies in existing oral PrEP services (eg, specific needles) and the availability of HCPs trained to administer the CAB-LA injections:

I think one of the biggest barriers could be related to specific health service supplies, such as the appropriate size of the needle - we don’t have all needle sizes available in a regular oral PrEP service (PrEP-SUS). Usually, in STI treatment cases, if the patient can opt to have their medication on the arm, they will take it, but on the butt only if they must, as with Penicillin. So, I think few qualified professionals would be available to administer the deep intramuscular injection.

Cisgender men, aged 40 to 45 years

The presence of peer educators at the clinics as an enabler of greater hospitality and person-centered approach was raised as an important facilitating factor for effective uptake of CAB-LA PrEP services. Due to their bond with potential users of PrEP, peer educators and navigators were believed to play a key role in reaching, approaching, and supporting participants interest in CAB-LA PrEP, particularly those under higher vulnerability to HIV:

I think one barrier in real-world, on a day-to-day basis, would be to improve the flow and include injectable PrEP procedures along with other activities. A facilitator, community education and peer support, but I don’t know if all services will provide them. I think that community education, a peer, someone who plays this role is important, especially at the beginning, as we don’t know how the general acceptance for the extensive HIV screening and the PrEP injections will be. Someone they can identify with for support will be helpful.

Transgender woman, aged 40 to 45 years

Participants expressed concerns about the adequacy of care for SGM and emphasized the need for training HCPs on how to appropriately approach this population, including the importance of using individuals' chosen names and respecting their gender identities. Finally, stigma and resistance to new health technologies by HCPs has been seen as a possible barrier for the implementation of injectable PrEP.

#### Feedback on the mHealth Tool

Overall, FGD participants believed the mHealth tool in video format would be very useful in supporting eligible users of PrEP in choosing between oral and injectable PrEP. They valued character representativeness in the mHealth tool, for example, by the presence of a transgender woman as physician and a Black gay man as the host, but suggested the inclusion of additional characters with other gender identities:

I like, the format, the colors, the presence of a transgender physician and the gay presenter as well. Having SGM representation in the videos is important for people to identify themselves with the service offered.

Cisgender woman, aged 50 to 55 years

Regarding the length of the mHealth tool, participants emphasized the importance of keeping videos concise and focused on the essential information for users’ choice, including PrEP modalities, STI screening, amount of time required for the clinical visits; and the periodicity of injectable PrEP appointments to manage participants’ expectations:

I don’t know if it’s worth reinforcing the side effects both PrEP may have. Some were included, but when they talk about choosing oral or injectable PrEP, I think we should explain more. Also, explain they will collect other STI tests, and when will be the time for returning appointments, for example: “Oh, you’ll come back in 30 days, than in 60 days, and if something happens and you can’t return, try to contact the health service for orientation.”

Transgender woman, aged 40 to 45 years

HCPs perceived that the videos should also include information on postinjection care and explain the possibility of CAB-LA injection interruption due to adverse events. They raised concerns that some potential users would need reassurance about the efficacy and duration of protection of both PrEP modalities and suggested including information about the efficacy of CAB-LA PrEP and the duration of protection against HIV. Likewise, they suggested including information on the possibility of switching prevention modalities from CAB-LA to oral PrEP and vice versa:

I think we should talk about side effects and switching, reinforce the positive aspects of using injectable cabotegravir because we had some volunteers from the previous clinical trial who ended up choosing to interrupt the injections. They did not tolerate the pain and chose to stop the injections. So, maybe the videos ought to talk about the advantage of having more than one prevention method available. I think there is the possibility of the person saying that they did not adapt [to oral/injectable PrEP] and want to switch.

Cisgender man, aged 50 to 55 years

Incorporating information about postexposure prophylaxis and the situations in which it can be used, such as after unprotected sex, sexual violence, or work accidents, was also recommended allowing the viewer a more comprehensive understanding of the available HIV prevention strategies.

As a final point, while participants anticipated the mHealth tool’s usefulness and practicality, they also asserted the importance of having face-to-face counseling with a health professional about PrEP modalities. They believed that specific technical, clinical, and adherence information regarding injectable PrEP is most effectively conveyed by an HCP. In addition, they emphasized the importance of reinforcing the message that participants should feel at ease to decide about their PrEP choice without feeling rushed. Having trained HCPs to clarify and reassure potential users regarding possible side effects was underscored.

#### Acceptability of WhatsApp Text Reminder Messages

There was consensus among HCPs participating in FGDs that using text messages for appointment reminders and support would be an acceptable form of communication to users of PrEP. Text messages were seen as more practical and reliable than phone calls to reach patients, allowing them to better manage their time for injectable PrEP consultations in advance. Moreover, there was a clear preference for the WhatsApp platform over other text message platforms due to its versality and familiarity among the Brazilian SGM populations:

I think it is convenient to receive reminders of consultation appointment with date and time directly on the mobile phone, which is a device present in everyday life for most people. It may improve adherence by ensuring patients are reminded to attend the necessary follow-up appointments for cabotegravir injections.

Cisgender woman, aged 45 to 50 years

### SGM Groups

#### Perceptions About Implementation of Injectable PrEP

SGM persons participating in FGDs believed that injectable PrEP would provide a convenient alternative to oral PrEP for people who have difficulty in taking daily medication or keeping their pills at home or who do not wish to use oral PrEP for other reasons. However, there were expressed concerns with the absence of specialized health care units:

I think injectable PrEP is more discreet. And I think people feel less exposed, right? Because the 18-year-old guy, who lives with his parents, has that bottle of pills there and the parents are going to see it. I think it’s more discreet than the pills in relation to stigma and discrimination, and social fear. So, I think that’s a benefit.

Cisgender MSM, aged 23 years

Due to the possible long duration of the initial visit for injectable PrEP initiation, SGM participants perceived that potential users of CAB-LA should be informed beforehand, so they have a more realistic expectation of the length of time needed. In addition, community education and peer educators were considered important to ensuring a welcoming, comprehensive health service adapted to the reality of users. Because of their connection to the SGM community, peer educators and navigators were perceived as playing a key role in reaching, approaching, contacting, and maintaining retention of users to PrEP injection visits. In addition, the SGM participants mentioned the importance of disseminating information about HIV prevention options and services, including injectable PrEP. They highlighted that information should be delivered in spaces other than the health care environment, such as NGOs and community venues, perceived as a more inclusive approach to reach SGM persons who are under higher vulnerability to HIV, such as those living in the outskirts and engaging in sex work.

Stigma and discrimination, common concerns of SGM populations who use health services, were also expressed for the implementation of injectable PrEP—among them were lack of knowledge and sensibility of how to properly refer to SGM populations. Resistance from HCPs to using an SGM individual’s chosen name could create an uncomfortable and disrespectful environment for those people seeking injectable PrEP. Possible stigmatization associated with moral judgment of sexual behaviors of persons using injectable PrEP was denoted as having a negative impact on their dignity, self-esteem, and persistence on PrEP services:

It’s just that it looks like there’s...a resistance of the professional to call the person by the chosen name, when the person doesn’t correct them.

Transgender woman, aged 26 years

And when we talk about our sexual practices they [HCP] come with a huge load of moralism. So that to me is something that just pushes me away and makes me not being adherent regardless of how good the drug is.

Cisgender MSM, aged 24 years

Provision of injectable PrEP without judgment or discrimination was strongly emphasized as a requirement for respectful and effective care. Participants indicated the necessity for capacity building of HCPs on gender identity and diversity to promote a sensitive and welcoming environment in injectable PrEP services. According to them, HCPs from these services should be empathetic, inclusive, and respectful of the diversity of people’s gender identities and sexual orientations to facilitate a trusting relationship with the client. The absence of gender and sexual diversity among HCPs was identified as a contributing factor to the inadequate care of these populations, possibly impacting initial choice and retention to injectable PrEP service.

#### Feedback on the mHealth Tool

In general, SGM persons participating in FGDs perceived the mHealth tool verbal and esthetic languages as appealing, accessible, direct, and relevant to reaching younger SGM groups. Some transgender and nonbinary participants thought that the videos could use more colloquial language for easier understanding. They emphasized the importance of avoiding stereotypes, using neutral language and inclusive pronouns, and respect for gender identities. Overall, participants thought that the use of digital tools for PrEP education combined with standard counseling would be very useful for participants to obtain a better understanding of PrEP modalities and make informed choices:

I think the use of the video in a tablet to explain injectable PrEP is doable! It reduces the time of the HCP to provide basic information, and the professional will have more time to discuss our doubts and worries. He/she takes that time, you know, to talk about, other subjects. It’s a very easy technology and tool, in this case in our favor, right? I think it’s useful.

Cisgender MSM, aged 20 years

The characters chosen for the videos were considered correct by SGM participants. Transgender and nonbinary participants suggested adding transgender men and nonbinary characters for greater diversity and to make the messaging more inclusive. Finally, they suggested including the credentials for the transgender woman physician to emphasize her credibility.

The mHealth tool was considered visually appealing. The use of images, colors, and drawings was praised as a joyful and simplified way to convey information and facilitate understanding. The use of electronic sounds or music was perceived as unpleasant and disturbing for most participants, although the younger SGM participants found the voice amplifying effects novel and flashy and thought they might increase the viewer’s attention. The insertion of subtitles and a sign language interpreter in the videos was highlighted as very important for accessibility and inclusivity, particularly for people with hearing impairment and in noisy environments:

The caption makes a lot of difference in a video. Especially when you’re in a bus, on a crowded bus and you can’t hear. As the colleague said, we follow through the caption. So, the caption makes a lot of difference.

Cisgender MSM, age 19 years

SGM participants considered the length of the videos appropriate. They suggested a clearer division of the introductory contents and other specific more in-depth videos on PrEP and emphasized the importance of a linear and organized approach in line with the proposed prototype. Different opinions about the order of the videos were expressed, with some people suggesting that the last video on HIV combination prevention should appear first. Participants recommended making the animations and colors more attractive. They also thought the videos should be more dynamic and less “professorial” in appearance and recommended keywords or summaries to reinforce the main information. The animated illustration of PrEP use schedule was praised for well-explaining the step-by-step procedures for the participant after choosing a PrEP modality (injectable or oral), including the reading of the informed consent:

Speaking of the video, the presentation of the graphic visuals. I liked the colors, it’s very harmonious, the video was dynamic because we have this exchange, this chatter, so the gay man asks the questions, and she [transgender woman physician] answers. It is very well explained, the answer is very clear to those who watch the video, the main issues are described in detail in the video. And so, it makes you really understand and see what the initial process is like.

Cisgender MSM, aged 29 years

Although participants considered the content of the videos to be very good, they pointed out additional information needs. They requested information about injectable PrEP for people with silicone implants and the impact and interaction of CAB-LA with hormone therapy. They also advised further explanation of the difference between daily and on-demand oral PrEP and the advantages and possible side effects of each. Concerning duration of PrEP protection, they pointed out the need to clarify (1) the reason for the interval between CAB-LA injections; (2) how to proceed if the user forgets a dose of daily oral PrEP; (3) the importance of daily oral PrEP adherence; and (4) the possibility of switching between PrEP modalities and potential consequences of switching, emphasizing the intervals needed and the waiting time for the new PrEP choice to provide protection:

My question is, if you start with oral PrEP...I have no problem taking medicine if I’m sick and all that. But my routine is quite busy too, so at some point I might forget. So, the question is, if I start on oral PrEP and then I want to switch to injectable, is it possible?

Transgender woman, aged 30 years

Participants thought the mHealth videos should thoroughly address information regarding HIV combination prevention, including the use of condoms, lubricants, postexposure prophylaxis, and regular HIV testing in conjunction with oral and injectable PrEP. Condom promotion has been recognized as essential for preventing other STIs and should be reinforced. Transgender participants particularly suggested the inclusion of information about specialized services in transgender care.

#### Acceptability of WhatsApp Text Reminder Messages

All SGM participants agreed that receiving reminders to confirm appointments through WhatsApp would be helpful and facilitate communication with the health service. They called attention to the importance of providing relevant information such as the date, time, and purpose of the visit. However, they raised concerns about privacy and suggested the messages be generic to avoid revealing personal information. They proposed the message include an icon or symbol associated with the health service, such as the study logo, so that recipients could easily identify the source of the message:

What I’m thinking is that you need to remind the person in a subtle way. So, like, ‘Oh, remember to visit us on such and such a date.’ And I don’t know if [the message could] include a symbol. I think the study logo, right? And we know what it is. Thus, a very simple and direct message is fine for me. You don’t expose, you don’t say what it is in detail, just communicate reminding for the appointment, you get the message[...].

Cisgender MSM, aged 20 years

There were different opinions on which messages would be the most appropriate. In the context of the study, participants expressed preference for reminder messages (“Don’t forget your return visit on that date”). They stressed the importance of using more discreet, general messages, avoiding keywords such as PrEP, injection, and HIV-related consultations to protect their privacy. They also emphasized the importance of personalizing reminder messages to increase effectiveness by adding a greeting, such as “Hello, so-and-so, don’t forget your visit, we are waiting for you.” They believed these types of messages would create a stronger connection and help people feel valued. There was a consensus that text messages are more practical and efficient than phone calls, and that they prefer short and direct messages that are easy to read and remember. There was a concern that SGM groups from underserved settings may have challenges reading long messages and that illiteracy may be an issue. Thus, they emphasized the need to approach communication in a careful and inclusive way.

## Discussion

### Principal Findings

This study describes findings from the formative work for ImPrEP CAB Brasil, the first study to evaluate CAB-LA PrEP implementation for young SGM groups in Latin America in a context of PrEP choice (CAB-LA or daily oral PrEP). The results of this study may be useful to aid researchers from Latin America and other regions for the preparation of CAB-LA implementation within their public health systems.

Formative work is a fundamental step to anticipate potential issues for injectable PrEP implementation and to provide critical information to tailor interventions to support PrEP acceptability, uptake, and choice. Our results revealed that an mHealth tool for PrEP education and WhatsApp text message reminders for injectable PrEP are highly acceptable and reinforced the likely benefit of using these tools in the implementation study. Recommendations regarding the implementation of injectable PrEP, refinement of the mHealth tool, and WhatsApp messages were considered and implemented before ImPrEP CAB Brasil study initiation.

Study participants believed that injectable PrEP provided a good alternative option to oral PrEP for those who have difficulty taking a daily pill. Recent studies among SGM groups from Brazil identified long-acting, injectable PrEP as preferred over daily oral PrEP [28,29]. However, knowledge on HIV prevention including PrEP remains limited among some population groups in Brazil, such as young SGM groups [30]. Dissemination of comprehensive information about PrEP modalities targeting young SGM groups in different formats and venues, including social media, is important to increase PrEP uptake and scale-up.

Consistent with prior studies, our results point to the need for training of HCPs on gender identity and diversity for effective PrEP implementation [31-33]. A recent survey conducted among SGM groups from Brazil showed that they experience high rates of discrimination in health care services [34]. In our study, SGM groups emphasized that a sensitive and respectful approach to SGM populations by HCPs is fundamental to ensuring population diversity and retention in care for populations seeking PrEP particularly in the case of intramuscular injectable formulations. Providing a welcoming environment and providing services to SGM groups with respect, dignity, and nondiscrimination were indicated as crucial facilitators to access and linkage to PrEP services. Previous studies conducted in Brazil with transgender women and young cisgender MSM on PrEP have also shown the importance of setting an LGBTQIAPN+ friendly clinic environment and having peer educators to support PrEP retention and adherence [5,35]. Regardless of the type of service, the quality of care provided highly influences health-seeking behavior [36]. For example, a previous multisite study of the Adolescent Trials Network showed that improved linkage to care in clinical settings for youth was achieved through the absence of stigma, formal and informal community relationships, and the presence of confidentiality and social support [37]. Similarly, having peer educators to support young SGM groups will be essential, particularly to guarantee emotional support.

Our results indicated that the inclusion of community mobilization from study inception allowed SGM groups to forefront their needs and preferences aligned with existing viable resources. Incorporating SGM persons as peer navigators and educators in the study clinics will create a person-centered health environment that emphasizes participant support and respect for their autonomy. This approach will enhance trust in study procedures and, consequently, increase the likelihood of successful implementation.

Finally, we found that process modeling was a powerful tool for planning this study within existing health services in the Brazilian public health system. As has been shown in other studies, process maps were useful in identifying likely challenges for study visits and prompted discussion on how to effectively mitigate them [38,39]. Continuous monitoring of the implementation of mapped processes will help identify and address further barriers and solutions to CAB-LA PrEP delivery.

This study has limitations. Formative studies are essential to provide information to implementation studies but do not provide evidence of real-world experiences after implementation. Although we included young SGM groups from 6 different large urban Brazilian cities mirroring the population of the ImPrEP CAB Brasil study, we may have not captured critical perspectives from SGM groups from other regions. Social desirability bias may have impacted participants’ responses in the focus groups. To mitigate this bias, we used highly trained and experienced social researchers as group facilitators. Participants were reminded that there were no right or wrong answers and were encouraged to provide straightforward feedback that would help us best improve our study and evaluate tools.

### Conclusions

Structured formative work with SGM community members and HCPs generated important refinements to context-specific materials and the launch of ImPrEP CAB Brasil in 6 public health clinics. An mHealth educational tool and WhatsApp text message reminders were highly acceptable by SGM groups and HCPs, indicating their potential to support PrEP choice and adherence. Stigma and discrimination against SGM groups and users of PrEP among HCPs are persistent barriers to overcome. Comprehensive evaluations are needed to measure the effectiveness of the mHealth tool for PrEP education and choice and WhatsApp appointment reminders for PrEP injection.

## Appendix

Multimedia Appendix 1 Part of the process map for the initial pre-exposure prophylaxis visit at the Institute of Infectious Diseases–Fiocruz site.

## Abbreviations

CAB-LA: long-acting, injectable cabotegravir
cisgender MSM: gay, bisexual, and other cisgender man who have sex with men
FGD: focus group discussion
HCP: health care provider
HPTN: HIV Prevention Trials Network
LGBTQIAPN+: lesbian, gay, bisexual, transgender, queer, intersex, asexual, agender, pansexual and nonbinary persons
mHealth: mobile health
NGO: nongovernmental organization
PrEP: pre-exposure prophylaxis
SGM: sexual and gender minority
STI: sexually transmitted infection
SUS: Sistema Único de Saúde
