# Supplementary figures and images for: Preparing for the Implementation of Long-Acting Injectable Cabotegravir for HIV Pre-Exposure Prophylaxis Within the Brazilian Public Health System (ImPrEP CAB Brasil): Qualitative Study
Source: JMIR Public Health Surveill. 2024 Oct 24;10:e60961. doi: 10.2196/60961 (PMC11544328; doi:10.2196/60961)

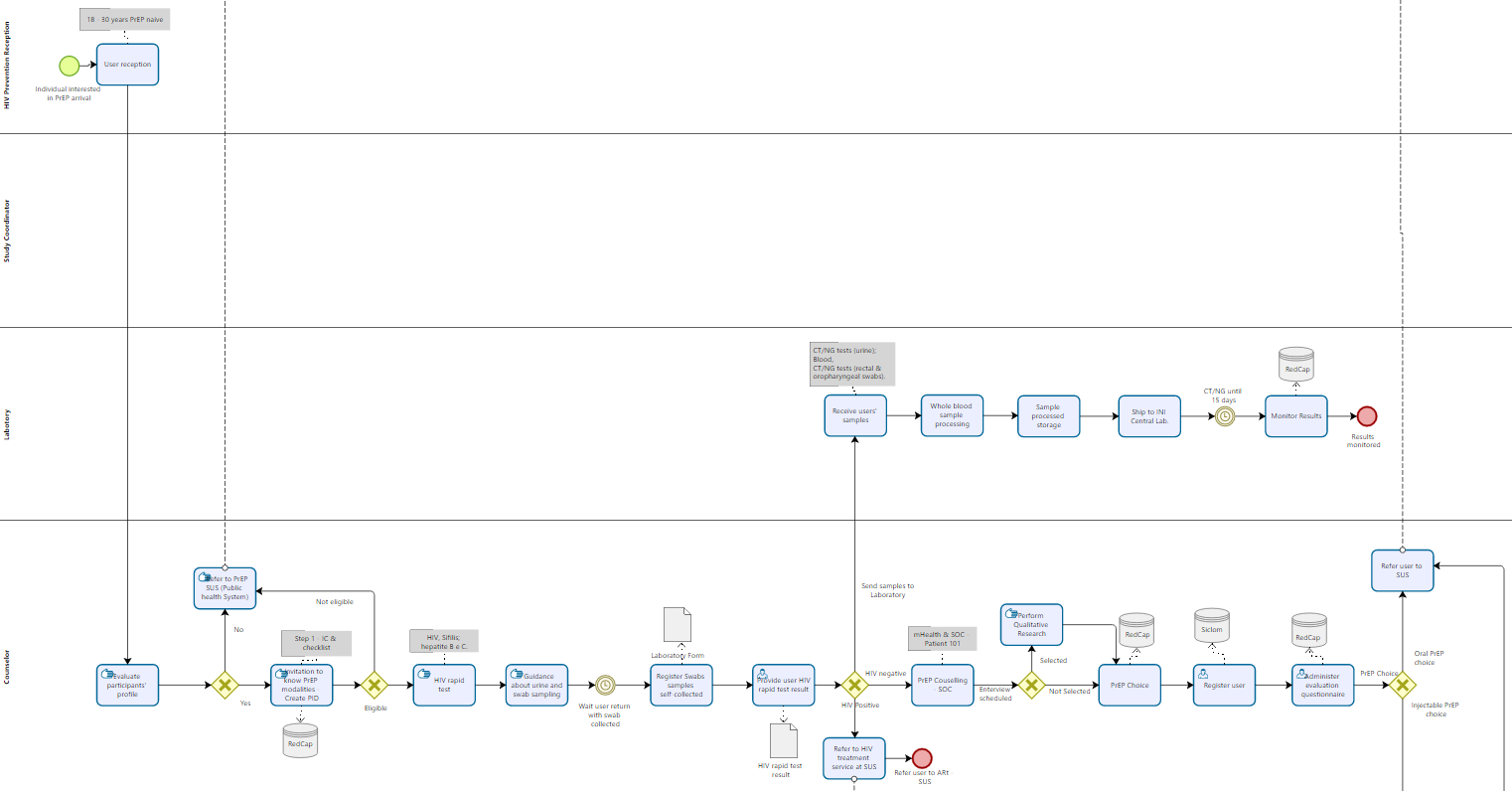

Supplement: Multimedia Appendix 1 [file publichealth_v10i1e60961_app1.png]
